# Educational Attainment and Diabetic Foot Ulceration: Outcomes From the Barbados Diabetic Foot Study

**DOI:** 10.1111/iwj.70969

**Published:** 2026-06-05

**Authors:** Laura Nicole Lovell, Nachiappan Chockalingam, Nina Davies, Peter Chami, Natalie S. Greaves

**Affiliations:** ^1^ Faculty of Medical Sciences The University of the West Indies Bridgetown Barbados; ^2^ University of Staffordshire Staffordshire UK; ^3^ Reading Central PCN ‐ NHS England Reading UK; ^4^ Leeds Community Healthcare NHS Trust Leeds UK; ^5^ Faculty of Science and Technology The University of the West Indies Bridgetown Barbados

**Keywords:** afro‐Caribbean, diabetes, diabetic foot, disparity, educational attainment, foot ulcer, healing outcomes, SINBAD, small island developing state

## Abstract

Diabetic foot ulceration (DFU) contributes significantly to diabetes‐related morbidity and amputation. In Barbados, where amputation rates are among the highest globally, the influence of socioeconomic factors on ulceration outcomes remains underexplored. Educational attainment, a social determinant of health, may influence health behaviours, engagement with healthcare services, and ultimately clinical outcomes. This study examines whether educational attainment is associated with diabetic foot ulcer severity, as measured by the SINBAD scoring system, and six‐week healing outcomes among inpatients with DFU. A prospective observational study was conducted over 6 months at Barbados' sole public hospital. A total of 176 participants admitted with a diagnosis of DFU were recruited. Baseline demographics, comorbidities, and ulcer characteristics were collected, and SINBAD scores were determined. Random forest modelling was employed to evaluate predictors of complete healing at 6 weeks and to assess ulcer severity stratified by educational attainment. Of the cohort, 17.5% reported primary education as their highest attainment level, compared with 2.9% of the general adult population. The mean SINBAD score was 2.45 among those with primary education and 2.51 among those with secondary education (*p* > 0.05). No statistically significant association was found between educational attainment and healing outcomes at 6 weeks. Educational attainment in this inpatient DFU cohort was lower than that of the general Barbadian population; however, it was not significantly associated with ulcer severity or six‐week healing outcomes. In a universal healthcare setting, equitable access to care may attenuate the effect of educational attainment on clinical outcomes. These null findings highlight the need for future adequately powered studies incorporating health literacy assessment and key clinical confounders. Nonetheless, the observed disparity in educational attainment among DFU inpatients suggests that foot health education initiatives should be designed to be accessible to individuals across all educational levels.

## 1 Introduction

Diabetic Foot Ulceration (DFU) is a complication of diabetes for which the lifetime risk is 19%–34% among individuals with the condition [1]. The prevalence of diabetes in the Caribbean region is increasing at an alarming rate [2], with approximately one in seven adults living with diabetes [3]. Barbados is an Eastern Caribbean small island developing state (SIDS) where estimated rates of diabetes are 13.2% in the adult population [4] and 42.5% of the inpatient population in a 2014 study [5]. Diabetic foot disease (DFD) contributes substantially to the clinical burden, accounting for 30% of all admissions and 89% of all diabetes‐related admissions [5] in the same 2014 account. DFD is also a personal burden to many individuals with psychosocial challenges and a strain on an individual's support systems [6]. Burdens on social structures are inherently linked to social determinants of health.

### 1.1 Social Determinants of Health

Social determinants of health have been shown to impact the likelihood of development of DFU as well as the outcome [7]. In a UK population‐based study in 2021, patients of the most deprived Townsend quintile had an increased risk of diabetic foot disease (DFD) compared with those of the least deprived after adjustment for other factors such as age, sex, and cardiovascular disease [8]. Outcomes such as increased mortality have also been noted in socially deprived groups [7]. While previous studies have linked DFU development to social deprivation, including financial insecurity and areas of residence [9], these influences are complex and multi‐factorial.

### 1.2 Education and the Social Determinants of Health

Multiple frameworks for understanding social determinants, including the WHO Commission's conceptual framework [10], the Dahlgren‐Whitehead model of health determinants [11] and the life course epidemiology perspective [12], position educational attainment as a structural determinant that influences health through multiple downstream pathways include income, occupation, and health literacy. Educational attainment or the ability of a person within society to receive schooling [13], as a social determinant of health, offers a distinctive lens for examining both early‐life and long‐term socioeconomic influences. Educational attainment reflects access and opportunity during childhood and adolescence and shapes later engagement with employment, income, and health behaviours [10]. Education has been identified as a “neglected social determinant of health,” and may affect how individuals interpret health promotion messages, engage in self‐care, and navigate healthcare systems [14]. Importantly, education may also affect how individuals interpret and act on health promotion messages and engage with healthcare services [10]. A 2026 review also highlighted that persons with low educational levels were more likely to have DFUs, and this may be a potential barrier to providing information in traditional methods [15].

Other SDHs which have been shown to affect DFUs may include geographical remoteness and access to health services [16]. However, in the context of a Barbadian health system, these may not be as pronounced given the smallness of the society and free public healthcare. In health systems where care is fee based, disparities in healthcare between those of higher and lower deprivation are well established; but in Caribbean communities with a Beveridge model of healthcare, provision of universal healthcare and access is less an issue [9]. Under this system, healthcare is publicly funded through taxation and available to all citizens regardless of income or occupation [17]. Nonetheless, challenges, such as long waiting times, persist [17].

### 1.3 Healthcare Disparities With Equal Access

In the UK, where a Beveridge taxation model also exists, people in areas of deprivation have higher healthcare need and delayed engagement with health systems [18]. In Barbados, disparities may be linked to economic pressures such as time off work (linked to employment level), health literacy gaps and systemic strain (e.g., wait times and limited outreach). Despite the provision of universal healthcare and education, disparities from other factors in a SIDS may affect the prevalence and outcomes of DFU. One such factor, educational attainment, has not been previously evaluated in SID health systems with universal healthcare through Beveridge funding.

Educational attainment in the Barbadian population is near universal as well; with 97% of citizens enrolled in primary education for persons aged 4 to 11 (kindergarten to 6th Grade) and 91% in secondary education for ages 11 to 16 years (6th grade to 11th grade) [19]. Education through to university level is also publicly funded through the government and is compulsory upto the age of 16 years. For context, Barbados spends about 5% of its GDP on education, boasting of a near universal literacy rate of over 96% driven by equitable access to empower a “knowledge‐based economy” [20]. Regarding educational attainment [19], in the 2021 census, the highest attainment of education in the 20–24 age group was secondary level (60.7%), with 17.9% achieving post‐secondary education (e.g., associate degree) and 19.3% receiving tertiary education (e.g., undergraduate degree) [21]. In the 30 and over group 9.6% of residents received primary education as their highest level, with 60.3% and 19.9% receiving secondary and tertiary as their highest levels, respectively [21].

However, in a universal healthcare setting, the impact on education may not have a uniform impact throughout a health system. Free education was established in Barbados in 1962 after the abolishment of fees at public secondary schools [22]. It is useful to understand this in the context of education and health, while related have a complex relationship as persons across different generations may have unique experiences while all being part of a singular society [23]. Equitable access to education may modulate the effect of universal healthcare in some settings. However, within Barbados access to free education up to age 16 years is available for all citizens, virtually negating this risk of access. However, we acknowledge that capturing the educational attainment of a cohort of mixed generations is observational, with many causes for terminating education before the accessible age of 16 years.

DFU is still a complication of increasing concern despite access to healthcare and education. Given Barbados' combination of universal education and universal healthcare yet persistently high rates of diabetic foot disease, this study aims to explore the relationship between educational attainment and DFU outcomes among inpatients. Specifically, we examine whether educational attainment is associated with (1) DFU severity as measured by the SINBAD classification system, and (2) healing outcomes at 6 weeks post‐admission. We hypothesise that, in the context of universal healthcare access, educational attainment would not be significantly associated with ulcer severity or short‐term healing outcomes, as equitable access to care may mitigate the effect of this social determinant.

## 2 Materials and Methods

### 2.1 Study Design

This was a prospective observational cohort study conducted at the sole public hospital in Barbados over a six‐month period (1 January 2024 to 30 June 2024).

### 2.2 Ethics Statement

Institutional review board approval (IRB No. CREC‐CH.00124/11/2022) from the University of the West Indies, Cave Hill Campus as well as the Queen Elizabeth Hospital, Barbados was obtained for this study.

### 2.3 Study Setting

The study was conducted within the Accident and Emergency Department of the public hospital of Barbados during the period 1 January 2024 to 30 June 2024. Persons accessing care at the hospital may be referred by their physician or can self‐refer to the department. All new admissions with a documented diagnosis of DFU were eligible for inclusion. Participants were identified through their admission diagnosis; no additional screening for DFU was conducted among the broader inpatient population. This approach captured individuals presenting specifically with DFU as a primary or contributing diagnosis requiring inpatient management.

### 2.4 Data Collection

New admissions were interviewed by trained data collectors after informed consent. Participant demographics (e.g., age, sex, income, highest level of formal education attained), medical comorbidities (such as hypertension, cardiovascular disease and dyslipidemia), diabetes complications (e.g., nephropathy, retinopathy) and ulcer characteristics (ulcer staging using SINBAD criteria), time from symptom onset to presentation in the emergency department, were collected using MAGPI software. Educational attainment was categorised according to local census categories by highest level of schooling completed: none, primary, secondary, high school or baccalaureate or technical or commercial career, bachelor's degree (undergraduate degree) or postgraduate degree [21]. Contact information for all participants was stored for recall at 6 weeks post‐admission.

### 2.5 Outcome Measures

The primary outcomes were: (1) DFU severity as measured by the SINBAD score, stratified by educational attainment; and (2) complete ulcer healing at 6 weeks post‐admission. SINBAD (Site, Ischemia, Neuropathy, Bacterial infection, Area, Depth) is a validated scoring system for classifying DFU severity, with scores ranging from 0 to 6, where higher scores indicate greater ulcer complexity. After discharge from the study site, all patients are followed up for a standard of six weeks within the Barbadian health system as normative practice. Care after discharge is fragmented (public polyclinics, private facilities), making longer‐term tracking challenging with a higher likelihood of attrition. In this context, as all patients are seen at six weeks, this represents a pragmatic time point at which participants return for hospital review. Six‐week recall evaluated continued hospitalisation, ulcer outcome (healing or non‐healing), amputation (major or minor), and new diagnosis of diabetes complication (such as retinopathy or nephropathy).

### 2.6 Data Analysis

Baseline demographics were described by means (SD) and frequencies (%) as appropriate. Group differences (between Low SINBAD and high SINBAD scores) were tested using Chi [2] tests. Educational attainment distribution was compared with national census data using chi‐square goodness‐of‐fit testing.

Random forest modelling was applied to identify predictors of complete healing at 6 weeks, including educational attainment, age, comorbidities, and SINBAD score. Random forest modelling was selected for its capacity to assess non‐linear relationships and variable importance across multiple predictors without assuming a specific distributional form. Additionally, SINBAD scores were compared across educational attainment levels. Analyses were conducted using STATA [24] and R [25].

Inpatient DFU incidence over the six‐month study period was also estimated by dividing the total number of patients admitted with a diagnosis of DFU by the estimated number of persons living with diabetes (PLWDs) in the Barbadian population, annualised over the 0.5‐year study period. This represents a hospital‐based incidence rate and should not be interpreted as population‐based incidence, as it captures only individuals seeking care at the public hospital.

Missing data were characterised as both missing at random (MAR) and missing not at random (MNAR). Multiple imputation assuming MAR and sensitivity analyses to test the robustness of findings under MNAR scenarios were employed.

## 3 Results

### 3.1 Baseline Demographics

176 participants were recruited during the study period with an admission diagnosis of DFU. The mean age of the population was 63.4 ± 11.2 years, with males accounting for 55.7% of the population and 77.8% identified as Christian religion. Time from symptom onset to first presentation in the emergency department was within 2 days for 58% of participants. Inpatient DFU incidence was also calculated at 0.23% over the study period.

SINBAD criteria were also determined for ulcerations of all participants with the mean SINBAD score of 2.4 (low risk score) and the median score of 2. The most common comorbidity in the study group was hypertension as shown in Table 1 below.

**TABLE 1:** Baseline characteristics of the study population.

Characteristics	All	Low SINBAD score	High sinbad score	*P*	Chi^2^
Age	63.4 ± 11.2			0.047	
Sex					
Sex, female (*n*%)	78 (44.3)	63 (80.7)	15 (19.2)		2.052
Sex, male (*n*%)	98 (55.7)	70 (71.4)	28 (28.6)		
Income (gross annual pay)					
0–49 000	65 (36.9)	49 (75.4)	16 (0.25)		12.815
50 000–99 000	5 (2.84)	4 (80.0)	1 (20.0)		
Over 99 000	1 (0.57)	0	1 (100)		
None	19 (10.8)	18 (94.7)	1 (5.3)		
Do not wish to respond	9 (5.11)	9 (100)	0		
Welfare support or NIS benefit	72 (40.9)	49 (68.1)	23 (31.9)		
Other	5 (2.8)	2 (40.0)	3 (60.0)		
Comorbidities at baseline					
Hypertension	104 (59.1)	78 (75.0)	26 (25.0)		0.045
Dislipidemia	35 (19.9)	25 (71.4)	10 (28.6)		0.406
Ischemic heart disease	12 (6.8)	8 (66.4)	4 (33.3)		0.553
Heart failure	8 (4.6)	6 (75)	2 (25.0)		0.002
Arrythmia	4 (2.3)	3 (75.0)	1 (25.0)		0.001
CVA or TIA event	6 (3.4)	3 (50.0)	3 (50.0)		2.2
Minor amputation history	41 (23.3)	23 (56.1)	18 (43.9)		10.976
Major amputation history	11 (6.3)	6 (54.5)	5 (45.5)		2.809

Abbreviations: CVA, cerebrovascular accident; NIS, national insurance service; SINBAD, Site, Ischemia, Neuropathy, Bacterial infection, Area, Depth; TIA, transient ischaemic attack.

### 3.2 Educational Attainment

Most participants completed secondary education as their terminal education (56.8%), with very few pursuing university‐level education (1.7%). The majority were also in the lower income band, with 40.9% of them on state benefits (NIS benefit) and welfare support. This is compared with 19% of the adult population (over 18 years) in Barbados as shown in Table 2. The distribution of educational attainment in the study cohort differed markedly from the general adult population, as shown in Table 2. Notably, 17.5% of study participants reported primary education as their highest attainment, compared with only 2.9% of the general adult population aged over 18 years. Chi‐square goodness‐of‐fit testing confirmed that the educational distribution of the study cohort differed significantly from the general population (*χ* ^2^ = 132.4, *p* < 0.001).

**TABLE 2:** Highest educational level of BDFS study cohort and total over 18 population.

	Highest educational level (*n* %)				
	Total population	Primary school	Secondary school	High school/technical/commercial	University education
Total population (over 18) (2021 census) [21]	269 090	7911 (2.9)	51 019 (19.0)	15 145 (5.8)	19 495 (7.2)
Barbados diabetic foot study participants	176	30 (17.5)	100 (56.8)	43 (24.4)	3 (1.7)
*χ* ^2^ Goodness‐of‐fit		*p* < 0.001			

It is important to note that the mean age of participants was 63.4 years, meaning that their educational experiences would reflect the educational landscape of Barbados approximately 40–50 years ago, rather than the current census data. While the 2021 census provides the most recent population‐level comparator, differences in educational opportunity across generations should be considered when interpreting this comparison.

Low rates of post‐secondary attainment were seen, with only three participants in the cohort achieving university education as shown in Table 3. There was no statistical difference for any educational attainment level despite this.

**TABLE 3:** Educational attainment and SINBAD scoring of participants.

Ulcer classification (*n* %)				
Highest educational level	Low SINBAD score	High SINBAD score	Chi^2^	Pr
Primary school education (*n* = 30)	21 (70.0)	9 (30.0)	0.61	0.436
Secondary school (*n* = 100)	73 (73.0)	27 (27.0)	0.83	0.363
High School/technical/commercial Career (*n* = 43)	36 (83.7)	7 (16.3)	2.05	0.152
University education (*n* = 3)	1 (33.3)	2 (66.6)	2.95	0.086

*Note:* At the 6‐week recall, 12 persons were identified as “healed 100%” with an attrition of 8% as shown in Table 4.

**TABLE 4:** Healing rate and attrition.

	All	Healed rate (less attrition)
Healed 100% at 6 weeks	12	7.14%
Attrition rate	8.0	

Missing data were characterised both by missing at random (MAR) and missing not at random (MNAR) patterns. Multiple imputation assuming MAR and sensitivity analyses to test the robustness of findings under MNAR scenarios were employed to address this.

### 3.3 SINBAD Modelling and Schooling

**FIGURE 1:**
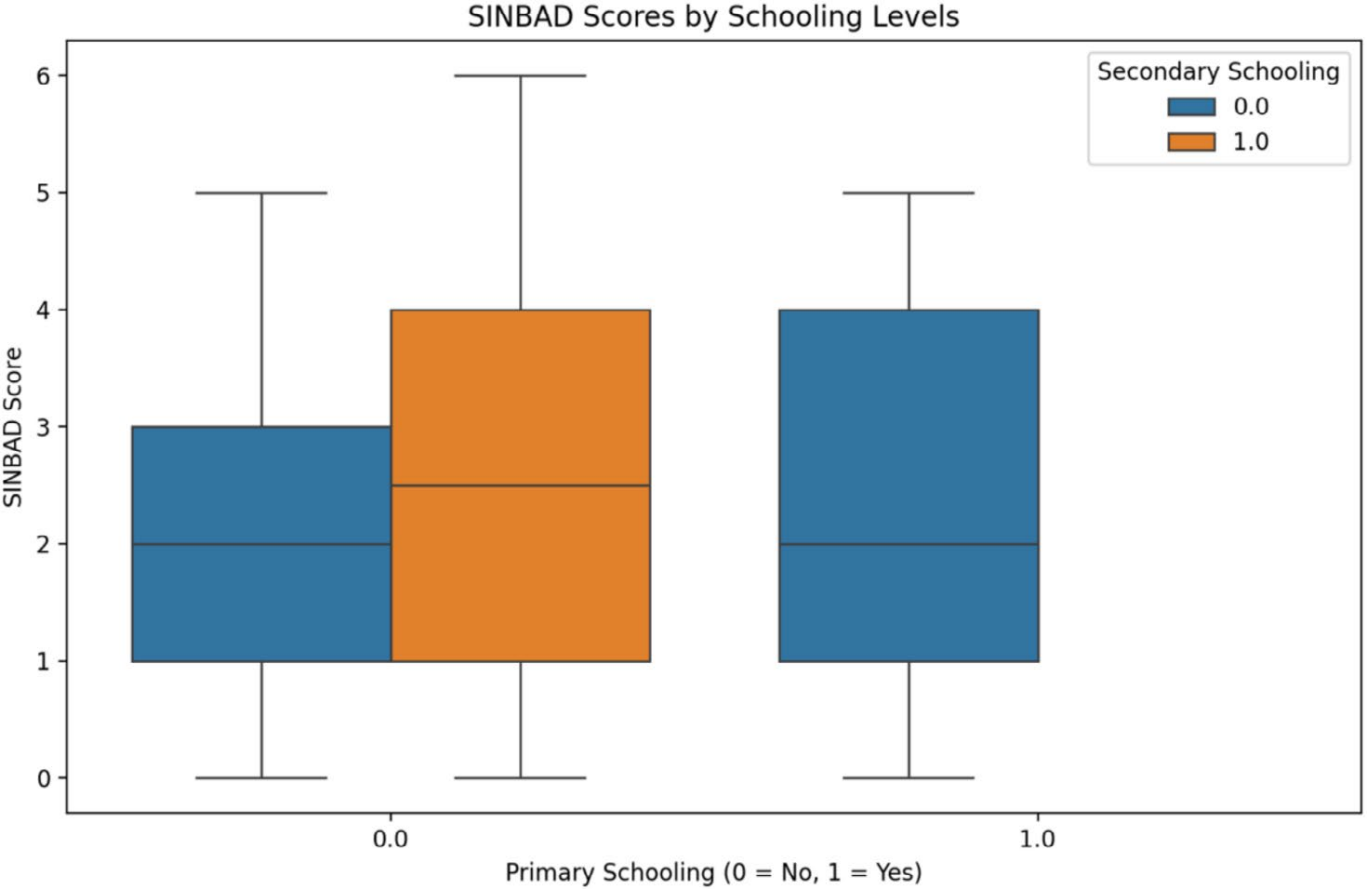
Healing outcome at 6 weeks (box and whisker plot).

The model achieved optimal accuracy in predicting complete healing outcomes on the test set. The feature importance rankings (in order) were age (0.045), ischemic heart disease (0.169), SINBAD score (0.147), hypertension (0.096), and retinopathy (0.065). Modelling for SINBAD scored by schooling levels shows that primary education only shows a mean SINBAD score of 2.45, whereas secondary education shows a mean SINBAD score of 2.51 in Figure 1. The difference is not statistically significant, suggesting education level may not strongly influence ulceration outcome.

## 4 Discussion

This study examined the association between educational attainment and DFU outcomes among inpatients in Barbados, a SIDS with universal healthcare and near‐universal education. Despite having a notably lower educational attainment than the general population, the principal findings showed that educational attainment was not significantly associated with ulcer severity (SINBAD score) or six‐week healing outcomes. These findings are discussed in the context of Barbados' unique healthcare and educational landscape.

### 4.1 Educational Disparity in the DFU Cohort

A key observation was the marked disparity in educational attainment between the study cohort and the general population. While only 2.9% of the adult Barbadian population reports primary education as their highest attainment, 17.5% of DFU inpatients were in this category. This overrepresentation of lower educational attainment among DFU inpatients is consistent with the broader literature linking socioeconomic disadvantage to diabetes complications [26]. However, the interpretation of this finding requires caution, given the mean age of participants (63.4 years), whose educational opportunities would have reflected the Barbadian educational landscape of the 1960s–1970s rather than current enrolment rates. Historical data on educational attainment specific to this generation were not available for direct comparison.

### 4.2 The Role of Universal Healthcare

The absence of a significant association between educational attainment and ulcer severity or healing outcomes may reflect the protective effect of Barbados' universal healthcare system. Under the Beveridge model, all citizens have access to hospital‐based care regardless of income or educational background. This contrasts with findings from fee‐based health systems, where lower educational attainment is more consistently associated with poorer wound outcomes due to barriers in accessing care. In Barbados, once patients are admitted, they receive standardised acute care, which may attenuate the influence of upstream social determinants on short‐term clinical outcomes.

This interpretation aligns with findings from other universal healthcare systems. In the UK, while social deprivation is associated with increased incidence of DFD, the association between deprivation and acute outcomes is less clear once patients are within the healthcare system [8, 12]. The current study's null finding may therefore represent a genuine attenuation of the education–outcome relationship within a universal access framework, rather than simply a failure to detect an association.

### 4.3 Hospital‐Based Incidence

The hospital‐based DFU incidence of 0.23% over the study period is notably lower than might be expected given the high burden of DFD previously reported in Barbados [27]. Several factors may contribute to this apparent discrepancy. During the study period, repeated patient surge notices were issued by the hospital, discouraging potential patients from seeking care due to overburdened emergency services secondary to trauma and non‐communicable disease burden. This may have diverted patients with DFU to primary care polyclinics or private hospitals, resulting in underreporting of true population‐level incidence. The hospital‐based incidence reported here should therefore be interpreted with caution and is not generalizable to population‐based incidence.

### 4.4 Implications for Foot Health Education

While this study did not measure health literacy directly, the observed disparity in educational attainment within the DFU cohort warrants consideration for clinical practice. In Barbados, health promotion initiatives for diabetes management are typically designed for a secondary school reading level. The finding that 17.5% of DFU inpatients had only primary‐level education suggests that some patients may face challenges engaging with standard health education materials. This aligns with findings from another Barbadian study that identified literacy barriers as a potential obstacle to effective patient education in diabetes management [13].

However, this study cannot establish a causal link between educational attainment and health behaviours or outcomes. The absence of health literacy measurement is a significant limitation, as educational attainment is an imperfect proxy for health literacy [25]. Individuals with lower formal educational attainment may possess considerable practical health knowledge acquired through lived experience, while individuals with higher educational attainment may still have poor health literacy in specific domains.

### 4.5 Comparison With Existing Literature

The null findings of this study are somewhat contrary to the broader literature, which generally supports an association between lower educational attainment and poorer DFU outcomes. Several factors may explain this discrepancy. First, the universal healthcare setting in Barbados may reduce the impact of educational attainment compared with mixed or private systems. Second, the study may have been underpowered to detect a clinically meaningful difference given the sample size and low event rate. Third, the relatively compressed range of educational attainment in this cohort, with 74.3% having secondary or lower education, may have limited the capacity to detect an education gradient. Future research in comparable SIDS settings would be valuable to determine whether these findings are replicable.

## 5 Study Limitations and Strengths

This study has several limitations that should be considered. First, no formal power calculation was performed a priori. With 176 participants and only 12 healing events at 6 weeks, the study is likely underpowered to detect clinically meaningful differences in healing outcomes by educational attainment. The null findings should therefore be interpreted as an absence of evidence rather than evidence of absence.

Second, the six‐week follow‐up period is shorter than the 12‐week endpoint recommended by international guidelines for assessing DFU healing. Most DFUs do not heal within 6 weeks, with 30%–40% achieving healing at 12 weeks. The short follow‐up was selected pragmatically to minimise attrition, given the fragmented nature of post‐discharge care in Barbados, but it may have limited the ability to observe meaningful differences in healing trajectories.

Third, important clinical confounders were not included in the analysis. HbA1c levels, wound care protocols, offloading adherence were not systematically captured. However, this study was observational and HbA1c is only performed on a limited basis in the public healthcare system. Care was not standardised across the cohort, and variation in treatment could independently influence healing outcomes. SINBAD scores reflect ulcer severity at presentation, which is itself influenced by multiple clinical factors beyond educational attainment and thus this study appropriately represented current clinical context.

Fourth, health literacy was not directly measured. Educational attainment serves as an imperfect surrogate for health literacy, and the absence of a validated health literacy assessment limits the ability to draw conclusions about the mechanism through which education might influence DFU outcomes.

Fifth, the granularity of educational attainment categories, while aligned with national census definitions, may have contributed to small subgroup sizes, particularly at the university level (*n* = 3). Sixth, the comparison with 2021 census data for a cohort whose educational experiences occurred 40–50 years ago introduces potential historical bias.

Finally, recruitment from the public inpatient population introduces selection bias. Individuals who sought care at private facilities may differ systematically in educational attainment and income, limiting the generalizability of findings to the broader DFU population.

Despite the absence of a statistically significant association, the marked disparity in educational attainment between the DFU cohort and the general population has practical implications for clinical care. Health promotion initiatives for DFD should be designed to be accessible to individuals with at least a primary (Grade 6) level of terminal education, incorporating visual aids and plain language approaches alongside written materials.

Strengths of this study include its prospective design, use of a validated ulcer classification system (SINBAD), systematic data collection using digital tools, and its focus on an underrepresented SIDS population. The handling of missing data through multiple imputation with sensitivity analyses is also a methodological strength. To our knowledge, this is the first study to examine educational attainment and DFU outcomes in a Caribbean SIDS with universal healthcare.

## 6 Conclusion

No statistically significant association was found between educational attainment and DFU severity or six‐week healing outcomes in this Barbadian inpatient cohort. The universal healthcare context may attenuate the influence of educational attainment on acute DFU outcomes. However, the study was likely underpowered, and the null findings should be interpreted cautiously.

Future research should aim to address the limitations identified in this study. Specifically, adequately powered multicentre studies incorporating validated health literacy assessments, standardised wound care protocols, and longer follow‐up periods (12 weeks or more) are needed to more definitively assess the role of educational attainment in DFU outcomes. Inclusion of key clinical confounders such as HbA1c, offloading adherence, and time to initial presentation would strengthen the analysis. Comparative studies across SIDS with similar universal healthcare models would help determine whether the null findings observed here are specific to Barbados or generalizable across similar settings. However, findings of this study highlight the need for greater understanding of other clinical variables and their impact on DFU outcomes and the importance of adapting health promotion initiatives for various educational levels.

## Funding

The authors have nothing to report.

## Ethics Statement

Institutional review board approval (IRB No. CREC‐CH.00124/11/2022) from the University of the West Indies, Cave Hill Campus, as well as the Queen Elizabeth Hospital, Barbados were obtained for this study.

## Consent

All participants provided informed consent prior to enrollment in the study.

## Conflicts of Interest

The authors declare no conflicts of interest.

## Data Availability

The data that support the findings of this study are available from the corresponding author upon reasonable request.
